# Fragment size and level of cell-free DNA provide prognostic information in patients with advanced pancreatic cancer

**DOI:** 10.1186/s12967-018-1677-2

**Published:** 2018-11-06

**Authors:** Morten Lapin, Satu Oltedal, Kjersti Tjensvoll, Tove Buhl, Rune Smaaland, Herish Garresori, Milind Javle, Nils Idar Glenjen, Bente Kristin Abelseth, Bjørnar Gilje, Oddmund Nordgård

**Affiliations:** 1 Department of Haematology and Oncology, Stavanger University Hospital, 4068 Stavanger, Norway; 2 Laboratory for Molecular Biology, Stavanger University Hospital, 4068 Stavanger, Norway; 3 Department of Gastrointestinal (GI) Medical Oncology, Division of Cancer Medicine, The University of Texas MD Anderson Cancer Center, Houston, TX USA; 4 Department of Oncology, Haukeland University Hospital, Bergen, Norway

**Keywords:** Cell-free DNA, cfDNA, cfDNA fragment size, Pancreatic cancer

## Abstract

**Background:**

It was recently demonstrated that the size of cell-free DNA (cfDNA) fragments that originates from tumor cells are shorter than cfDNA fragments that originates from non-malignant cells. We investigated whether cfDNA fragment size and cfDNA levels might have prognostic value in patients with advanced pancreatic cancer.

**Methods:**

Blood samples were obtained from patients with advanced pancreatic cancer, before (n = 61) initiation of chemotherapy and after the first cycle of chemotherapy (n = 39). Samples were separated with density centrifugation and plasma DNA was isolated. Mode cfDNA fragment size and cfDNA levels were then determined using a 2100 Bioanalyzer. A cohort of partially age-matched healthy volunteers (n = 28) constituted the control group.

**Results:**

Both a pre-treatment cfDNA fragment size of ≤ 167 bp (mode) and high pre-treatment cfDNA levels were associated with shorter progression-free survival (PFS) (p = 0.002 and p < 0.001, respectively) and overall survival (OS) (p = 0.001 and p = 0.001, respectively). Furthermore, multivariable Cox regression analyses demonstrated that pre-treatment cfDNA levels could independently predict prognosis for both PFS (HR = 3.049, p = 0.005) and OS (HR = 2.236, p = 0.028).

**Conclusion:**

This study demonstrates that cfDNA fragment size and cfDNA levels can be used to predict disease outcome in patients with advanced pancreatic cancer. The described approach, using a rapid, economic and simple test to reveal prognostic information, has potential for future treatment stratification and monitoring.

**Electronic supplementary material:**

The online version of this article (10.1186/s12967-018-1677-2) contains supplementary material, which is available to authorized users.

## Background

Previous studies have demonstrated that cell-free DNA (cfDNA) levels are elevated in blood from patients with cancer compared to the levels in blood from healthy individuals [1]. Moreover, high levels of cfDNA have been associated with poor survival in several cancers, including pancreatic cancer [2–4]. Using surrogate markers, such as point mutations [5–10], copy-number aberrations [11, 12], microsatellite alterations [13] and methylations [12], it has been possible to detect the fraction of cfDNA that originates from tumor cells. This fraction, better known as circulating tumor DNA (ctDNA), can be detected in most patients with cancer, and both the fraction of patients with detectable ctDNA and the levels of ctDNA in blood have been shown to increase with increasing tumor stages [5]. In recent years, ctDNA has also been demonstrated to provide prognostic value and monitoring potential for patients with different cancers, including patients with pancreatic cancer [6–10].

Studies of cfDNA in maternal plasma and transplant recipients have indicated that cfDNA is predominantly of hematopoietic origin, and that cfDNA from non-hematopoietic cells are shorter in size than hematopoietically derived cfDNA [14–16]. In accordance with these observations, studies on cfDNA from patients with cancer have demonstrated that the fragments of cfDNA that originates from tumor cells (i.e. ctDNA) are, in fact, shorter in size than fragments that originate from non-malignant cells [17–20]. Another study recently demonstrated that size-selection of smaller cfDNA fragments could be used to increase the amount of tumor-derived cfDNA fragments in cfDNA samples [20]. Given the reported shorter size of tumor-derived cfDNA fragments, we reasoned that increasing numbers of short cfDNA fragments might indicate higher fractions of tumor-derived cfDNA and thus, might reflect an increased tumor load. Accordingly, we hypothesized that a cfDNA fragment size analysis might hold prognostic value in cancer. The present study aimed to investigate the prognostic value of cfDNA fragment size in cancer. We employed simple laboratory techniques to determine the fragment size and total levels of cfDNA isolated from patients with locally advanced or metastatic pancreatic cancer.

## Materials and methods

### Patients and samples

Between September 2012 and July 2017, we included 61 patients with locally advanced (n = 6) or metastatic (n = 55) pancreatic cancer that were admitted to Stavanger University Hospital (SUH) and Haukeland University Hospital (HUH). Patients received first-line treatment with gemcitabine (SUH only), nab-paclitaxel plus gemcitabine (SUH only), or FOLFIRINOX (both centers). Peripheral venous blood samples (9 ml EDTA tubes) were drawn before initiation of chemotherapy (n = 61; denoted B1) and after the first cycle of chemotherapy (n = 39; denoted B2). Treatment response was defined with a standard disease evaluation of radiological images, based on the RECIST 1.1 criteria [21]. We also included a control group (median age = 54), which comprised 28 healthy individuals with no prior or current cancer diagnosis. Blood samples collected at SUH were processed within 2 h; samples collected at HUH were processed at SUH within 24 h, after overnight transport. All patients and healthy controls provided written informed consent to participate in the study. The project was approved by the Regional Committee for Medical and Health Research Ethics (REK-Vest 2011/475, REK-Vest 2013/1743).

### Isolation of cfDNA from plasma

Blood samples were separated with density centrifugation using Lymphoprep™ (Axis Shield) density gradient media, according to the manufacturer’s instructions. We used 4 mL (1–2 mL for the first 8 patients) of plasma (diluted 1:1 with 0.9% NaCl for the density gradient separation protocol) for isolating total cfDNA with the QIAamp Circulating Nucleic Acid kit (Qiagen), as described by the manufacturer. cfDNA was eluted in 50 µL of Buffer AVE (Qiagen) and stored at − 80 °C until further analysis.

### Determination of cfDNA fragment size and cfDNA level

The cfDNA fragment size and cfDNA level were determined for each sample with an Agilent 2100 Bioanalyzer and the Agilent High Sensitivity DNA chip, according to the manufacturer’s instructions. The fragment size of cfDNA was determined, with the Agilent 2100 Bioanalyzer software, and defined as the mode of the main peak (corresponding to one nucleosome plus linker, derived from apoptotic cells) in the electropherogram (Additional file 1: Figure S1). cfdna level were also determined with the software, calculated as the area under the main peak in the electropherogram (Additional file 1: Figure S1). Determination of cfDNA fragment size and cfDNA levels were performed by individuals blinded to clinical data.

### Statistical analysis

All statistical analyses were performed with IBM SPSS version 24.0 (http://www.spss.com/). All tests were two-sided, and p-values less than 0.05 were considered statistically significant. Missing data were automatically excluded from the analyses. Clinicopathological patient data were compared with the χ^2^ or Fisher’s exact test, for categorical data, and with the independent-samples t-test or Mann–Whitney U test for continuous data. The Mann–Whitney U test was also used to compare cfDNA fragment size and cfDNA levels between control and patient samples, and between centers. The Wilcoxon signed rank test or the sign test were used to compare samples taken before and after initiation of chemotherapy. Correlation tests were performed between cfDNA fragment size and cfDNA levels with the non-parametric Spearman’s rank correlation coefficient for the continuous variables, and Cohen’s Kappa coefficient for the categorical variables. Data from the two study centers were compared statistically (Additional file 1: Table S1 and Figure S2). We found significant differences between centers in first-line treatment and cfDNA fragment size.

Survival analyses were performed with Kaplan–Meier estimates, the log-rank test, and Cox proportional hazards regression. The primary endpoints were progression-free survival (PFS) and overall survival (OS). PFS was defined as the time between inclusion (or sampling date for the B2 sample) and progression or death due to any cause, when the patient died before evidence of progression was obtained. OS was defined as the elapsed time between inclusion (or sampling date for the B2 sample) and death due to any cause.

Univariable Cox regression analyses were used to investigate the effects of single variables on survival. We tested variables measured before initiation of chemotherapy, including the B1 cfDNA fragment size (both continuous and dichotomized), B1 cfDNA levels (both continuous and dichotomized), the combination of B1 cfDNA fragment size and B1cfDNA levels, carbohydrate antigen 19-9 (CA 19-9) status, study center, age, sex, tumor size, tumor location, T-stage, clinical stage, metastatic location, N-stage, Eastern Cooperative Oncology Group (ECOG) performance status, first line treatment, second line treatment, and prior anti-cancer surgery. We also tested selected variables 1–2 months after the start of chemotherapy: the B2 cfDNA fragment size (both continuous and dichotomized) and B2 cfDNA levels (both continuous and dichotomized).

Multivariable Cox regression modeling was also performed for selected variables, including the B1 cfDNA fragment size (dichotomized), B1 cfDNA levels (dichotomized), CA 19-9 status, study center, age, sex, tumor size, tumor location, clinical stage, ECOG performance status, first line treatment, and prior anti-cancer surgery. Several variables were excluded from the multiple regression model, due to associations with other markers, including the B1 cfDNA fragment size (continuous), B1 cfDNA level (continuous), the combination of B1 cfDNA fragment size and B1 cfDNA levels. Other variables were excluded due to missing data (T-stage, metastatic location, N-stage), or because they were not acquired at baseline (B2 cfDNA fragment size, B2 cfDNA levels, and second-line treatment). The multivariable analyses were performed with both forward and backward stepwise selection of covariates.

## Results

### Patient characteristics

The 61 patients included in this study had either locally advanced (n = 6) or metastatic tumors (n = 55). The cohort included a slight preponderance of men (n = 35, 57.4%), and the mean age was 64 years at diagnosis. Primary tumors were predominately located in the head of the pancreas (55.7%), and the median size was 37 mm. The first six (9.8%) patients included in the study received Gemcitabine monotherapy; the remaining patients received either FOLFIRINOX (n = 30, 49.2%) or nab-paclitaxel plus Gemcitabine (n = 25, 41%). All baseline patient characteristics and clinicopathological data are summarized in Table 1.

**Table 1:** Baseline characteristics of patients with pancreatic cancer

Variable	All patients (n = 61)
Median age	64 (41–81)
Sex	
Male	35 (57%)
Female	26 (43%)
Median tumor size (mm)	37 (11–100)
Median CA 19-9 levels	385.5 (5–102041)
CA 19-9	
< 37U/mL	13 (21%)
≥ 37U/mL	47 (77%)
Missing data	1 (2%)
Tumor location	
Pancreas head	34 (56%)
Pancreas body	4 (7%)
Pancreas tail	13 (21%)
Multiple	10 (16%)
T-stage	
T2	11 (18%)
T3	15 (25%)
T4	25 (41%)
TX	2 (3%)
Missing data	8 (13%)
Clinical stage	
Stage III	6 (10%)
Stage IV	55 (90%)
Metastatic location	
Liver	25 (41%)
Lung	4 (7%)
Multiple	16 (26%)
Other	6 (10%)
Missing data	10 (16%)
N-stage	
N0	14 (23%)
N1	25 (41%)
NX	21 (34%)
Missing data	1 (2%)
ECOG status	
0	13 (21%)
1	34 (56%)
2	10 (16%)
Missing data	4 (7%)
First-line treatment	
Gemcitabine	6 (10%)
FOLFIRINOX	30 (49%)
Nab-Paclitaxel +	25 (41%)
Gemcitabine	
Second-line treatment	
Yes	17 (28%)
No	44 (72%)
Prior anti-cancer surgery	
Yes	9 (15%)
No	51 (84%)
Missing data	1 (2%)

### Measurements of cfDNA fragment size and cfDNA level

**Fig. 1:**
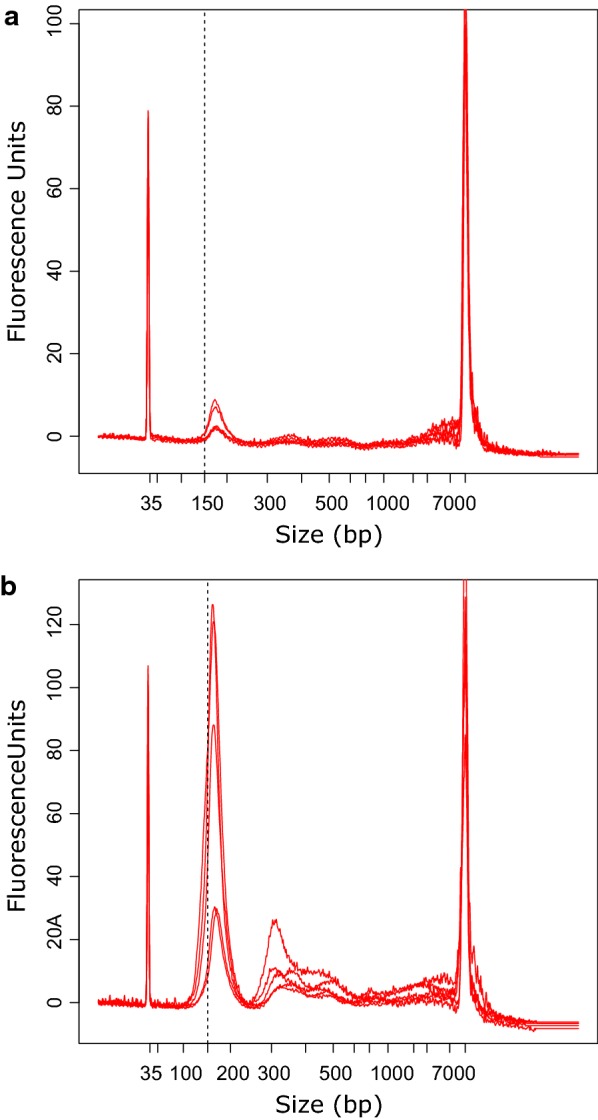
Distribution of cfDNA fragment size and cfDNA level in plasma. **a** cfDNA from healthy controls (n = 10) displayed larger fragment size (X-axis) and lower cfDNA level (Y-axis) compared to **b** cfDNA from patients with pancreatic cancer (n = 10). The dotted line placed at 150 bp indicates a shift towards shorter fragment size in patient samples

**Fig. 2:**
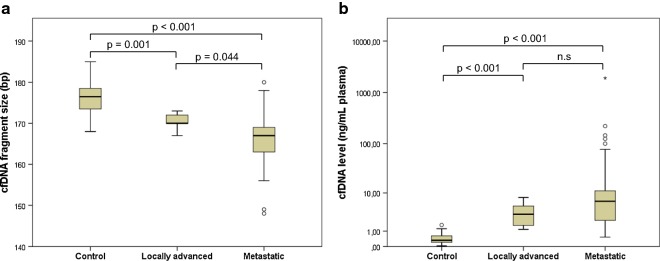
Distributions of cfDNA fragment size and cfDNA levels in patient and control samples. Boxplot shows differences in **a** cfDNA fragment size and **b** cfDNA levels between samples from healthy controls (n = 28), patients with localized disease (n = 6), and patients with metastatic disease (n = 55). Abbreviation: n.s, not significant

cfDNA fragment size and cfDNA levels were measured in plasma samples from the patients and from partially age-matched healthy controls (n = 28). The cfDNA fragment size in healthy control samples were longer (median 176.5 bp, range 168–185 bp) than the fragment size in patient samples (locally advanced: median 170 bp, range 167–173 bp, p = 0.001; metastatic: median 167 bp, range 148–180 bp, p < 0.001; Figs. 1 and 2). Moreover, the levels of cfDNA in healthy control samples (median 0.33 ng/mL plasma, range 0.03–1.61 ng/mL plasma) were significantly lower than those in patient samples (locally advanced: median 3.26 ng/mL plasma, range 1.16–7.98 ng/mL plasma, p < 0.001; metastatic: median 6.58 ng/mL plasma, range 0.53–1911.63 ng/mL plasma, p < 0.001; Figs. 1 and 2). When samples from patients with locally advanced tumors were compared to samples from patients with metastatic tumors, we found significant differences in cfDNA fragment size (p = 0.044; Fig. 2a), but not in cfDNA levels (p = 0.257; Fig. 2b).

### Changes in cfDNA fragment size and cfDNA levels during therapy

cfDNA fragment size and cfDNA levels were determined in plasma samples obtained before the initiation of chemotherapy (n = 61 patients), and in plasma samples obtained after the first cycle of chemotherapy (n = 39 patients). Neither the median cfDNA fragment (p = 0.956) nor the median cfDNA level (p = 0.110) changed significantly during treatment (Additional file 1: Figure S3).

### Survival analyses

**Fig. 3:**
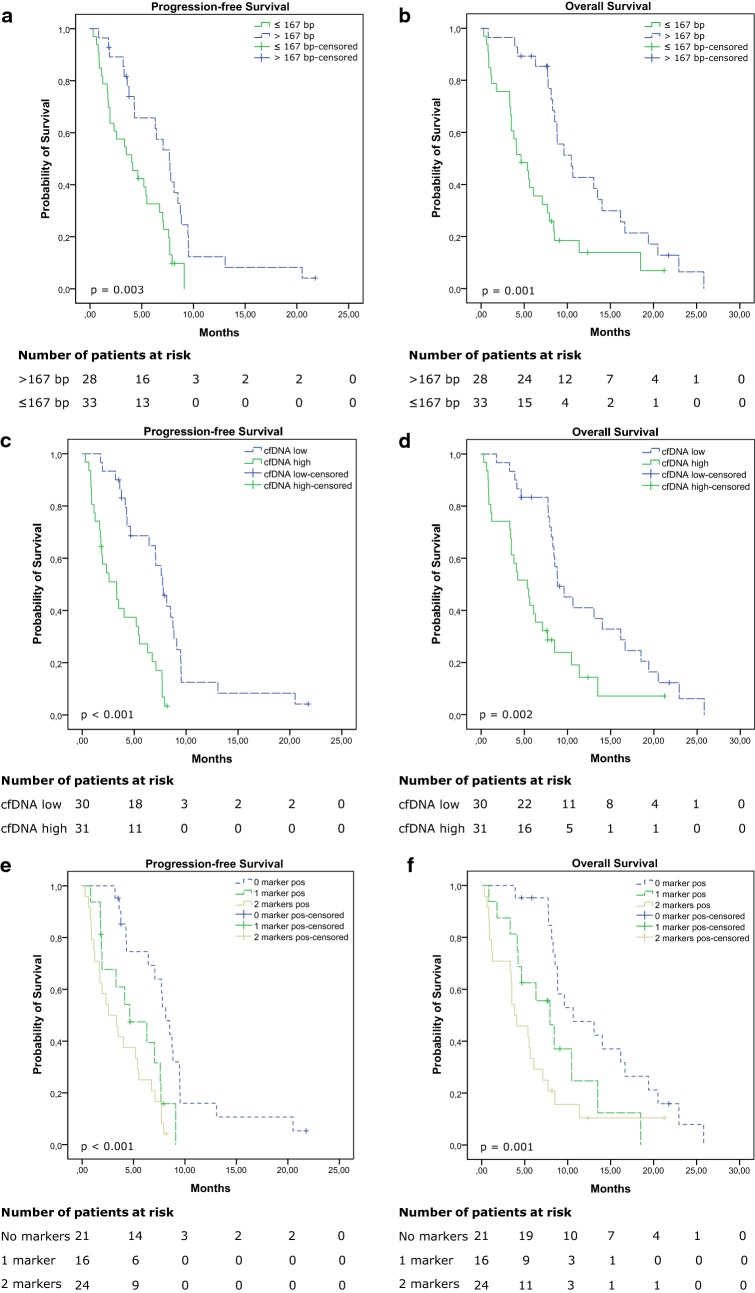
Kaplan-Meier analyses of pre-treatment samples. **a**, **c**, **e** Progression-free survival and **b**, **d**, **f** overall survival are shown for **a**, **b** patients with short (≤ 167 bp) vs those with long (> 167 bp) cfDNA fragment size; **c**, **d** patients with high vs those with low cfDNA levels, and **e**, **f** patients positive for two markers, vs those with one or no markers

Patients were followed for a median of 7.7 months (range 0.3–25.8 months). During follow-up, progression was observed in 54 (88.5%) patients, and 51 (83.6%) patients died. We dichotomized cfDNA fragment size and cfDNA levels in patient samples based on the median values of all patient samples; thus a short fragment size was defined as ≤ 167 bp, and a high cfDNA level was defined as > 4.66 ng/mL plasma. We found that a short pre-treatment cfDNA fragment size (≤ 167 bp) was highly associated with both shorter PFS (4.0 vs 7.7 months; log-rank p = 0.003; Fig. 3a) and shorter OS (4.6 vs 10.5 months; log-rank p = 0.001; Fig. 3b). Analyses of in-treatment cfDNA fragment size demonstrated that short fragment size were again associated with shorter PFS (3.1 vs 6.8 months; log-rank p = 0.049; Additional file 1: Figure S4A), but not with shorter OS (7.0 vs 7.0 months; log-rank p = 0.634; Additional file 1: Figure S4B). Similarly, patients with high pre-treatment cfDNA levels had both shorter PFS (3.3 vs 7.7 months; log-rank p < 0.001; Fig. 3c) and shorter OS (5.4 vs 8.8 months; log-rank p = 0.002; Fig. 3d) compared to patients with low cfDNA levels. In samples obtained after initiation of chemotherapy, high cfDNA levels were neither associated with shorter PFS (4.5 vs 6.9 months; log-rank p = 0.185; Additional file 1: Figure S4C), nor shorter OS (7.0 vs 7.4 months; log-rank p = 0.368; Additional file 1: Figure S4D).

We found a moderately negative correlation between pre-treatment cfDNA fragment size and cfDNA levels (spearman correlation − 0.608, p < 0.001). Short fragment size (≤ 167 bp) and high cfDNA levels (> 4.66 ng/mL plasma) were also concordant in 45/61 (74%; Kappa = 0.475; 95% CI 0.253–0.696) patients. To see if it provided additional prognostic value, we performed survival analysis on the combination of the pre-treatment cfDNA fragment size and cfDNA levels. The patients were grouped according to whether they had neither short cfDNA fragment size nor high cfDNA levels (negative for both markers), either short cfDNA fragment size or high cfDNA levels (positive for 1 marker), or both (positive for two markers). The survival analyses demonstrated that both PFS (8.1 vs 4.6 vs 2.6 months; log-rank p < 0.001; Fig. 3e) and OS (10.6 vs 7.9 vs 3.8 months; log-rank p = 0.001; Fig. 3f) were significantly prolonged in patients negative for both markers, compared to patients that were positive for one or two markers.

Both univariable and multivariable Cox proportional hazards regression analyses were performed to estimate the prognostic impact of cfDNA fragment size and cfDNA levels on survival, relative to other clinicopatological parameters. The univariable regression analyses (Table 2) confirmed the prognostic impact of cfDNA fragment size and cfDNA levels, as demonstrated by the Log-Rank test. In addition, the univariable analysis showed that radiologically determined tumor size, ECOG performance status, first-line treatment, second-line treatment and study center also had prognostic value. The multivariable Cox regression analyses (Table 3) demonstrated that the dichotomized cfDNA level was an independent prognostic factor for both PFS (HR = 3.049, p = 0.005) and OS (HR = 2.236, p = 0.028). In addition, CA 19-9 was an independent prognostic factor for OS. Finally, the ECOG performance status and first-line treatment were independent prognostic factors for both PFS and OS. No other covariates were identified as significant predictors of survival in the multivariable model.

**Table 2:** Univariable Cox regression

Parameter	Progression-free survival		Overall survival	
	Hazard ratio (95% CI)	p-value	Hazard ratio (95% CI)	p-value
cfDNA fragment size B1 (continuous)	0.913 (0.865–0.962)	*0.001*	0.897 (0.848–0.949)	*< 0.001*
cfDNA fragment size B1 (> 167 bp vs ≤ 167 bp)	0.409 (0.225–0.744)	*0.003*	0.394 (0.220–706)	*0.002*
cfDNA level B1 (continuous)	1.002 (1.001–1.003)	*0.001*	1.002 (1.001–1.003)	*0.001*
cfDNA level B1 (> median vs ≤ median)	3.773 (2.009–7.084)	*< 0.001*	2.418 (1.350–4.329)	*0.003*
Combination cfDNA fragment size and cfDNA levels		*< 0.001*		*0.002*
0 markers^a^	Reference		Reference	
1 marker	2.825 (1.275–6.258)		2.032 (0.943–4.379)	
2 markers	4.476 (2.139–9.364)		3.326 (1.695–6.526)	
CA 19-9 (< 37U/mL vs ≥ 37U/mL)	0.665 (0.319–1.385)	0.276	0.854 (0.412–1.771)	0.672
cfDNA fragment size B2 (continuous)	0.935 (0.860–1.017)	0.116	0.981 (0.899–1.070)	0.662
cfDNA fragment size B2 (> 167 bp vs ≤ 167 bp)	0.498 (0.245–1.011)	0.054	0.841 (0.413–1.715)	0.635
cfDNA levels B2 (continuous)	1.061 (1.028–1.095)	*< 0.001*	1.062 (1.026–1.099)	*0.001*
cfDNA levels B2 (> median vs ≤ median)	1.626 (0.786–3.365)	0.190	1.412 (0.664–3.002)	0.371
Age at diagnosis	1.003 (0.973–1.035)	0.833	0.990 (0.957–1.023)	0.539
Sex (male vs female)	1.194 (0.688–2.070)	0.529	1.194 (0.671–2.124)	0.546
Center (SUH vs HUH)	2.176 (1.153–4.105)	*0.016*	2.213 (1.130–4.337)	*0.021*
Tumor size (mm)	1.030 (1.012–1.049)	*0.001*	1.021 (1.001–1.041)	*0.042*
Tumor location		0.075		0.075
Pancreas head	Reference		Reference	
Pancreas body	0.616 (0.182–2.088)		0.657 (0.197–2.195)	
Pancreas tail	2.314 (1.165–4.596)		2.239 (1.125–4.458)	
Multiple	1.135 (0.531–2.425)		1.581 (0.732–3.434)	
T-stage		0.342		0.350
T2	Reference		Reference	
T3	1.116 (0.471–2.645)		0.922 (0.367–2.318)	
T4	0.664 (0.314–1.403)		0.603 (0.281–1.294)	
Clinical stage (Stage IV vs Stage III)	2.147 (0.836–5.515)	0.112	1.829 (0.715–4.678)	0.207
Metastatic location		0.457		0.735
Liver	Reference		Reference	
Lung	0.382 (0.113–1.288)		0.590 (0.195–1.781)	
Multiple	0.726 (0.271–1.944)		0.767 (0.262–2.240)	
Other	0.841 (0.439–1.610)		0.740 (0.366–1.496)	
N-stage (N1 vs N0)	1.745 (0.760–4.006)	0.189	1.566 (0.703–3.488)	0.273
ECOG performance status		*0.003*		*< 0.001*
0	Reference		Reference	
1	1.656 (0.809–3.389)		1.634 (0.782–3.415)	
2	5.057 (1.932–13.235)		6.810 (2.537–18.277)	
First-line treatment		*< 0.001*		*< 0.001*
FOLFIRINOX	Reference		Reference	
Gemcitabine	14.760 (4.874–44.697)		15.099 (4.951–46.047)	
Nab-Pac + gemcitabine	2.796 (1.481–5.280)		3.201 (1.665–6.155)	
Second-line treatment (yes vs no)	0.822 (0.453–1.490)	0.519	0.494 (0.255–0.955)	*0.036*
Prior anti-cancer surgery (yes vs no)	0.601 (0.287–1.260)	0.178	0.722 (0.324–1.612)	0.427

^a^ A positive marker indicates either short cfDNA fragment size or high cfDNA levels

Italic indicates a significant p-value

**Table 3:** Multivariable Cox regression

Parameter	Progression-free survival		Overall survival	
	Hazard ratio (95% CI)	p-value	Hazard ratio (95% CI)	p-value
cfDNA level B1 (> median vs ≤ median)	3.049 (1.398–6.649)	0.005	2.236 (1.089–4.590)	0.028
CA 19-9 (< 37U/mL vs ≥ 37U/mL)		n.s.	0.345 (0.148–0.804)	0.014
ECOG performance status		0.043		0.011
0	Reference		Reference	
1	1.122 (0.486–2.589)		1.149 (0.540–2.446)	
2	3.108 (1.066–9.056)		4.066 (1.443–11.461)	
First line treatment		0.003		< 0.001
FOLFIRINOX	Reference		Reference	
Gemcitabine	7.765 (2.313–26.068)		12.064 (3.385–42.996)	
Nab-Pac + gemcitabine	2.021 (1.002–4.076)		2.901 (1.411–5.965)	

*n.s.* not significant

## Discussion

In this study, we demonstrate that cfDNA fragment size and cfDNA level have prognostic value for patients with advanced pancreatic cancer. Previous studies have shown that increased levels of cfDNA measured before the initiation of chemotherapy are associated with worse survival in patients with pancreatic cancer [3] and with increased tumor load in patients with colorectal cancer [2]. Similarly to our approach, it was also recently demonstrated that shorter cfDNA fragment size are associated with shorter PFS in patients with renal cell carcinoma [22]. The fragment size of cfDNA that originates from non-hematopoietic cells [14–16], including tumor cells [19, 20], have been shown to be shorter than cfDNA that originates from cells of hematopoietic origin. That finding suggests that increased numbers of short cfDNA fragments primarily reflects increased relative levels of tumor DNA in the blood of patients with cancer. The mechanisms underlying the shorter fragment size of non-hematopoietically derived cfDNA are not well understood. However, it has been speculated that different cell types might have differences in intracellular DNA degradation and different mechanisms of DNA release, and that DNA methylation could be a contributing factor [16, 23].

Interestingly, our multivariable Cox regression model indicated that the cfDNA level was an independent prognostic factor, but not the cfDNA fragment size. Although cfDNA fragment size and cfDNA levels were moderately correlated in our cohort, we found that cfDNA fragment size was not an independent prognostic factor even when we tested cfDNA fragment size in the absence of cfDNA levels in the regression model (Additional file 1: Table S2). Nevertheless, it would be interesting to compare the levels of the tumor-derived cfDNA with cfDNA fragment size in future studies, to clarify the association between cfDNA fragment size and prognosis.

Both cfDNA fragment size and cfDNA levels were also measured 1 month after the initiation of chemotherapy. Previous studies have shown that patients that receive chemotherapy display significant increases in cfDNA levels at 24 h and 8 days after initiation of chemotherapy [24], and after several cycles of chemotherapy [25]. In this study, however, we did not observe any change in either cfDNA fragment size or cfDNA levels after treatment initiation. Further, we did not observe any association between cfDNA levels and survival after initiation of chemotherapy. In contrast, we did observe a weak association between cfDNA fragment size and PFS.

In this study, we found significant differences in the PFS and OS between the two study centers. The first six patients recruited at SUH were all treated with Gemcitabine monotherapy and died within 4 months after initiation of treatment. These patients were the only patients treated with Gemcitabine monotherapy, and their inclusion explained most of the difference in survival between the two centers. However, FOLFIRINOX treatment was given to a higher proportion of patients at HUH (100%) than at SUH (26%). In this study, FOLFIRINOX treatment was more beneficial for both PFS and OS than nab-paclitaxel treatment. The increase in survival among patients that received FOLFIRINOX compared to patients that received nab-paclitaxel could not be directly explained by differences in either ECOG performance status (p = 0.768) or clinical stage (p = 0.271). However, it should be mentioned that most patients that received FOLFIRINOX were recruited at HUH, and that we only recruited patients that were expected to tolerate FOLFIRINOX treatment from that center. Consequently, the patient selection method at HUH might have been biased. After we adjusted for covariates in the multivariable model, the study center was no longer an independent factor (Table 3).

This study had some limitations. First, we used the Agilent 2100 Bioanalyzer and Agilent High Sensitivity DNA kit to determine cfDNA fragment size and cfDNA levels. This kit was reported (from the manufacturer) to have a sizing accuracy of ± 10% coefficient of variation (CV) and a sizing reproducibility of 5% CV or better; moreover, its quantification accuracy was 20% CV and its quantification reproducibility was 15% CV or better. Thus, measurements close to the values of dichotomization might have represented false positives or false negatives. Alternatively, we might have obtained more precise cfDNA fragment size and cfDNA level estimates by utilizing next-generation sequencing (NGS) for size estimations [19, 20], and qPCR-based kits for quantification [19]. Higher precision might have increased the prognostic value of our markers. However, the Agilent 2100 Bioanalyzer is much simpler and faster than NGS and qPCR, and it provided reasonable accuracy and reproducibility. From a clinical perspective, a rapid, economic, simple test offers important advantages. However, it would be interesting, in future, to assess cfDNA size and cfDNA levels with more precise techniques to validate the current findings.

Another study limitation was the excess of patients with metastatic disease compared to patients with locally advanced disease, and the total lack of patients with localized disease. Consequently, our results are not broadly generalizable. Future studies should investigate whether cfDNA fragment size and cfDNA level also provide prognostic information for patients with resectable pancreatic cancer. It would also be of interest to investigate the prognostic potential of these markers in other cancer types. Most previous investigations of cfDNA in pancreatic cancer have focused on detecting mutated cfDNA, which show prognostic significance in patients with both resectable and unresectable pancreatic cancer [9, 26, 27]. Thus, a side-by-side comparison of the cfDNA fragment size and cfDNA level approach and the mutated cfDNA detection approach should be performed to investigate further the clinical potential of our findings.

## Conclusion

We demonstrated that the determination of cfDNA fragment size and cfDNA levels is a non-invasive, simple method for predicting clinical outcome in patients with advanced pancreatic cancer. Shorter cfDNA fragment size was associated with worse PFS and OS, although it was not an independent prognostic marker. In contrast the cfDNA level was a strong independent prognostic factor for both PFS and OS in our patient cohort. Future investigations are needed to elaborate these findings with regard to clinical relevance, and to establish whether the determination of cfDNA fragment size and cfDNA level might also have prognostic value for patients with localized pancreatic cancer.

## Additional file

**Additional file 1: Figure S1.** Patient sample electropherogram. Main peak corresponds to nucleosomes released from apoptotic cells (corresponding to one nucleosome plus linker). **Table S1.** Baseline characteristics of patients with pancreatic cancer. **Figure S2.** Comparison of cfDNA fragment size and cfDNA levels in plasma between centers. Boxplot showing differences in (A) cfDNA fragment size and (B) cfDNA levels between samples obtained at SUH versus samples obtained at HUH. **Figure S3.** Distributions of cfDNA fragment size and cfDNA levels in plasma at baseline and during treatment. Boxplot showing differences in (A) cfDNA fragment size and (B) cfDNA levels between samples obtained before initiation of chemotherapy versus samples obtained during treatment. **Figure S4.** Kaplan-Meier analyses of samples obtained after the first cycle of chemotherapy. (A, C) Progression-free survival and (B, D) overall survival are shown for (A, B) patients with short (≤167 bp) vs those with long (>167 bp) cfDNA fragment size, and (C, D) patients with high vs those with low cfDNA levels. **Table S2.** Multivariable Cox regression without cfDNA level.

## Abbreviations

cfDNA: cell-free DNA
PFS: progression-free survival
OS: overall survival
HR: hazard ratio
ctDNA: circulating tumor DNA
FOLFIRINOX: folinic acid, fluorouracil, irinotecan, oxaliplatin
CA 19-9: carbohydrate antigen 19-9
T-stage: tumor stage
N-stage: nodal stage
ECOG: Eastern Cooperative Oncology Group
