# Supplementary material for: Fragment size and level of cell-free DNA provide prognostic information in patients with advanced pancreatic cancer
Source: J Transl Med. 2018 Nov 6;16:300. doi: 10.1186/s12967-018-1677-2 (PMC6218961; doi:10.1186/s12967-018-1677-2)
Supplement: Supplementary file 1 — Additional file 1: Figure S1. Patient sample electropherogram. Main peak corresponds to nucleosomes released from apoptotic cells (corresponding to one nucleosome plus linker). Table S1. Baseline characteristics of patients with pancreatic cancer. Figure S2. Comparison of cfDNA fragment size and cfDNA levels in plasma between centers. Boxplot showing differences in (A) cfDNA fragment size and (B) cfDNA levels between samples obtained at SUH versus samples obtained at HUH. Figure S3. Distributions of cfDNA fragment size and cfDNA levels in plasma at baseline and during treatment. Boxplot showing differences in (A) cfDNA fragment size and (B) cfDNA levels between samples obtained before initiation of chemotherapy versus samples obtained during treatment. Figure S4. Kaplan-Meier analyses of samples obtained after the first cycle of chemotherapy. (A, C) Progression-free survival and (B, D) overall survival are shown for (A, B) patients with short (≤167 bp) vs those with long (>167 bp) cfDNA fragment size, and (C, D) patients with high vs those with low cfDNA levels. Table S2. Multivariable Cox regression without cfDNA level. [file 12967_2018_1677_MOESM1_ESM.docx]

**Fragment size and level of cell-free DNA provide prognostic information in patients with advanced pancreatic cancer**

Morten Lapin^1,2^, Satu Oltedal^1,2^, Kjersti Tjensvoll^1,2^, Tove Buhl^1,2^, Rune Smaaland^1,2^,

Herish Garresori^1^, Milind Javle^3^, Nils I. Glenjen^4^, Bente K. Abelseth^4^,

Bjørnar Gilje^1,2^, and Oddmund Nordgård^1,2^

1 Department of Haematology and Oncology, Stavanger University Hospital, N–4068 Stavanger, Norway.

2 Laboratory for Molecular Biology, Stavanger University Hospital, N–4068 Stavanger, Norway.

3 Department of Gastrointestinal (GI) Medical Oncology, Division of Cancer Medicine, The University of Texas MD Anderson Cancer Center, Houston, TX.

4 Department of Oncology, Haukeland University Hospital, Bergen, Norway.

| **Table S1 – Baseline characteristics of patients with pancreatic cancer** | | | | |
| --- | --- | --- | --- | --- |
| **Variable** | **All patients (n = 61)** | **SUH (n = 42)** | **HUH (n = 19)** | **p-value**^a^ |
| Mean age | 61 (41-81) | 63 (41-81) | 62 (46-72) | 0.530 |
| Sex |  |  |  | 0.956 |
| Male | 35 (57%) | 24 (57%) | 11 (58%) |  |
| Female | 26 (43%) | 18 (43%) | 8 (42%) |  |
| Median tumor size (mm) | 37 (11-100) | 36 (12-100) | 42 (11-98) | 0.970 |
| Median CA19-9 levels | 385.5 (5-102041) | 649 (5-102041) | 145 (5-73389) | 0.085 |
| CA19-9 |  |  |  | 0.552 |
| < 37U/mL | 13 (21%) | 8 (19%) | 5 (26%) |  |
| ≥ 37U/mL | 47 (77%) | 33 (79%) | 14 (74%) |  |
| Missing data | 1 (2%) | 1 (2%) | 0 (0%) |  |
| Tumor location |  |  |  | 0.913 |
| Head | 34 (56%) | 22 (52%) | 12 (63%) |  |
| Body | 4 (7%) | 3 (7%) | 1 (5%) |  |
| Tail | 13 (21%) | 10 (24%) | 3 (16%) |  |
| Multiple | 10 (16%) | 7 (17%) | 3 (16%) |  |
| T-stage |  |  |  | 0.055 |
| T2 | 11 (18%) | 10 (24%) | 1 (5%) |  |
| T3 | 15 (25%) | 7 (17%) | 8 (42%) |  |
| T4 | 25 (41%) | 16 (38%) | 9 (47%) |  |
| TX | 2 (3%) | 2 (5%) | 0 (0%) |  |
| Missing data | 8 (13%) | 7 (17%) | 1 (5%) |  |
| Clinical stage |  |  |  | 0.364 |
| Stage III | 6 (10%) | 3 (7%) | 3 (16%) |  |
| Stage IV | 55 (90%) | 39 (93%) | 16 (84%) |  |
| Metastases |  |  |  | 0.370 |
| M0 | 6 (10%) | 3 (7%) | 3 (16%) |  |
| M1 | 54 (89%) | 38 (91%) | 16 (84%) |  |
| MX | 1 (2%) | 1 (2%) | 0 (0%) |  |
| Metastatic location |  |  |  | 0.362 |
| Liver | 25 (41%) | 16 (38%) | 9 (47%) |  |
| Lung | 4 (7%) | 4 (10%) | 0 (0%) |  |
| Multiple | 16 (26%) | 14 (33%) | 2 (11%) |  |
| Other | 6 (10%) | 3 (7%) | 3 (16%) |  |
| Missing data | 10 (16%) | 5 (12%) | 5 (26%) |  |
| Lymph node status |  |  |  | 0.146 |
| N0 | 14 (23%) | 5 (12%) | 9 (47%) |  |
| N1 | 25 (41%) | 15 (36%) | 10 (53%) |  |
| NX | 21 (34%) | 21 (50%) | 0 (0%) |  |
| Missing data | 1 (2%) | 1 (2%) | 0 (0%) |  |
| ECOG status |  |  |  | 0.639 |
| 0 | 13 (21%) | 10 (24%) | 3 (16%) |  |
| 1 | 34 (56%) | 26 (62%) | 8 (42%) |  |
| 2 | 10 (16%) | 6 (14%) | 4 (21%) |  |
| Missing data | 4 (7%) | 0 (0%) | 4 (21%) |  |
| First-line treatment |  |  |  | **<0.001** |
| Gemcitabine | 6 (10%) | 6 (14%) | 0 (0%) |  |
| FOLFIRINOX | 30 (49%) | 11 (26%) | 19 (100%) |  |
| Nab-Paclitaxel + | 25 (41%) | 25 (60%) | 0 (0%) |  |
| Gemcitabine |  |  |  |  |
| Second-line treatment |  |  |  | 0.095 |
| Yes | 17 (28%) | 9 (21%) | 8 (42%) |  |
| No | 44 (72%) | 33 (79%) | 11 (58%) |  |
| Prior anti-cancer surgery |  |  |  | 0.581 |
| Yes |  | 7 (17%) | 2 (11%) |  |
| No |  | 35 (83%) | 16 (84%) |  |
| Missing data |  | 0 (0%) | 1 (5%) |  |
|  |  |  |  |  |
| ^a^Comparisons were performed between patients from the different centers. | | | | |
| **Boldface** indicates a significant p-value | | | | |

| **Table S2 - Multivariable Cox regression without cfDNA level** | | | | |
| --- | --- | --- | --- | --- |
| **Parameter** | **Progression-free survival** | | **Overall Survival** | |
|  | Hazard Ratio (95% CI) | p-value | Hazard Ratio (95% CI) | p-value |
| Median tumor size (mm) | 1.024 (1.005-1.043) | 0.015 |  | n.s. |
| ECOG performance status |  | 0.008 |  | 0.003 |
| 0 | Reference |  | Reference |  |
| 1 | 1.590 (0.710-3.559) |  | 1.374 (0.641-2.944) |  |
| 2 | 4.986 (1.724-14.416) |  | 5.199 (1.887-14.326) |  |
| First line treatment |  | <0.001 |  | <0.001 |
| FOLFIRINOX | Reference |  | Reference |  |
| Gemcitabine | 11.803 (3.685-37.807) |  | 17.564 (5.139-60.032) |  |
| Nab-Pac + gemcitabine | 2.100 (1.044-4.226) |  | 3.044 (1.472-6.298) |  |
|  |  |  |  |  |
| Abbreviation: n.s., not significant. |  |  |  |  |

**Figure S1: Patient sample electropherogram.** Main peak corresponds to nucleosomes released from apoptotic cells (corresponding to one nucleosome plus linker).


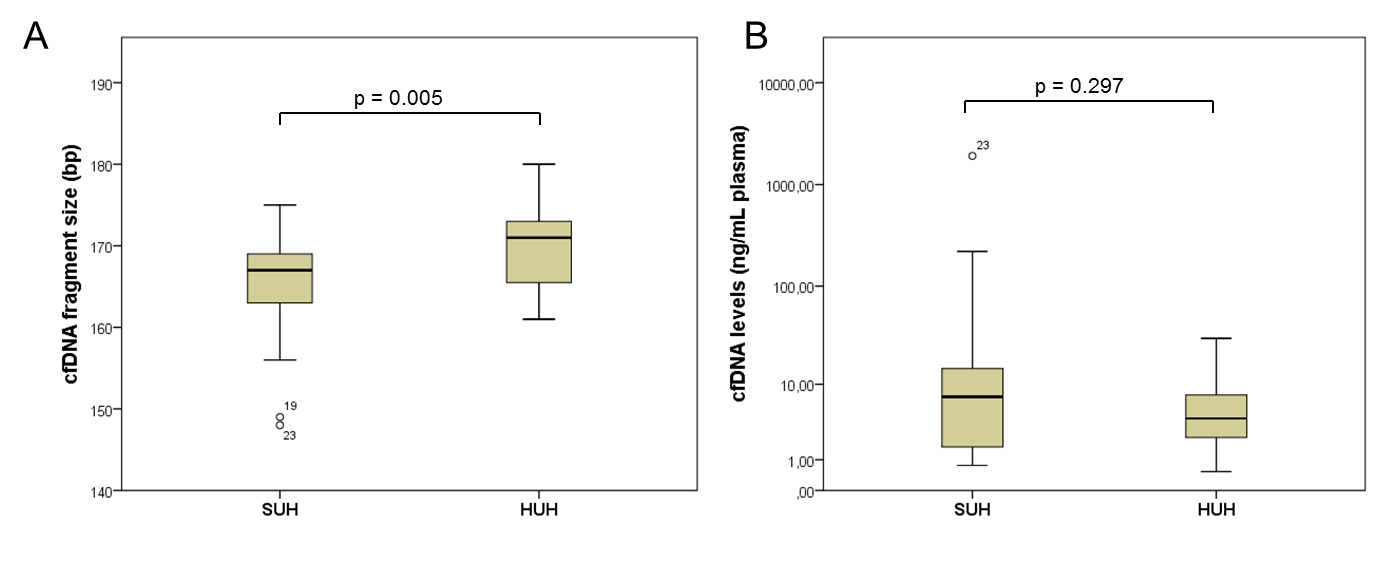


**Figure S2: Comparison of cfDNA fragment size and cfDNA levels in plasma between centers.** Boxplot showing differences in (**A**) cfDNA fragment size and (**B**) cfDNA levels between samples obtained at SUH versus samples obtained at HUH.

**
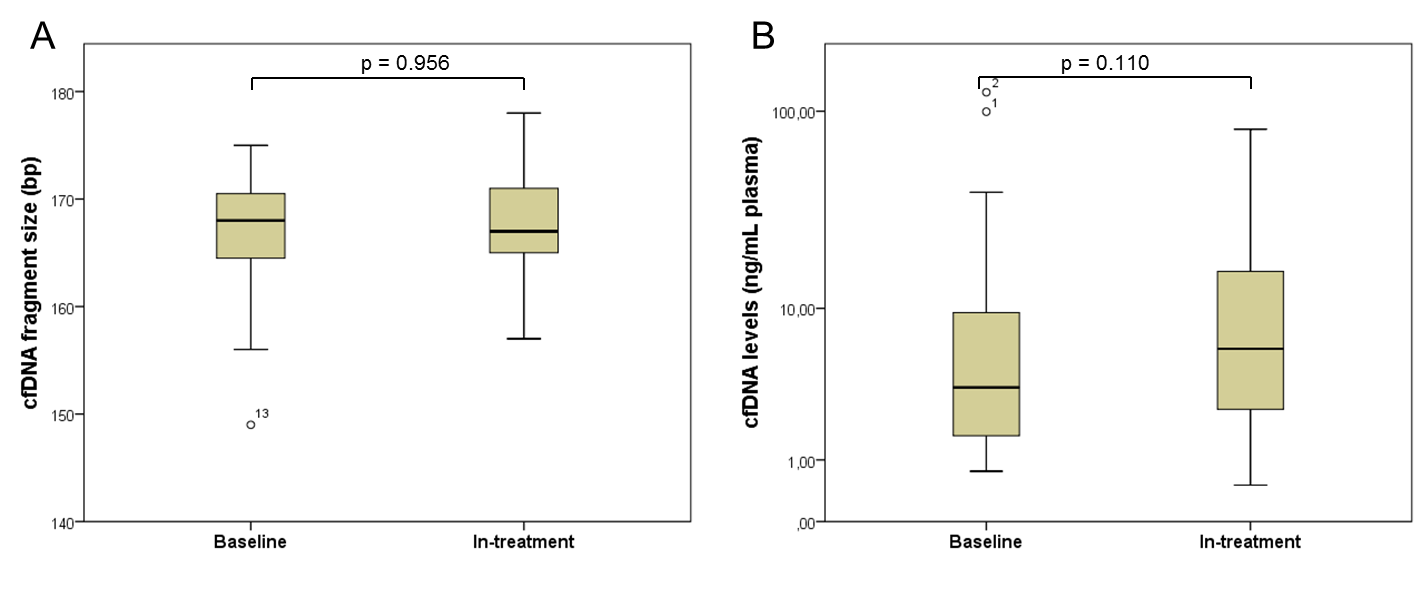
**

**Figure S3: Distributions of cfDNA fragment size and cfDNA levels in plasma at baseline and during treatment.** Boxplot showing differences in (**A**) cfDNA fragment size and (**B**) cfDNA levels between samples obtained before initiation of chemotherapy versus samples obtained during treatment.

**
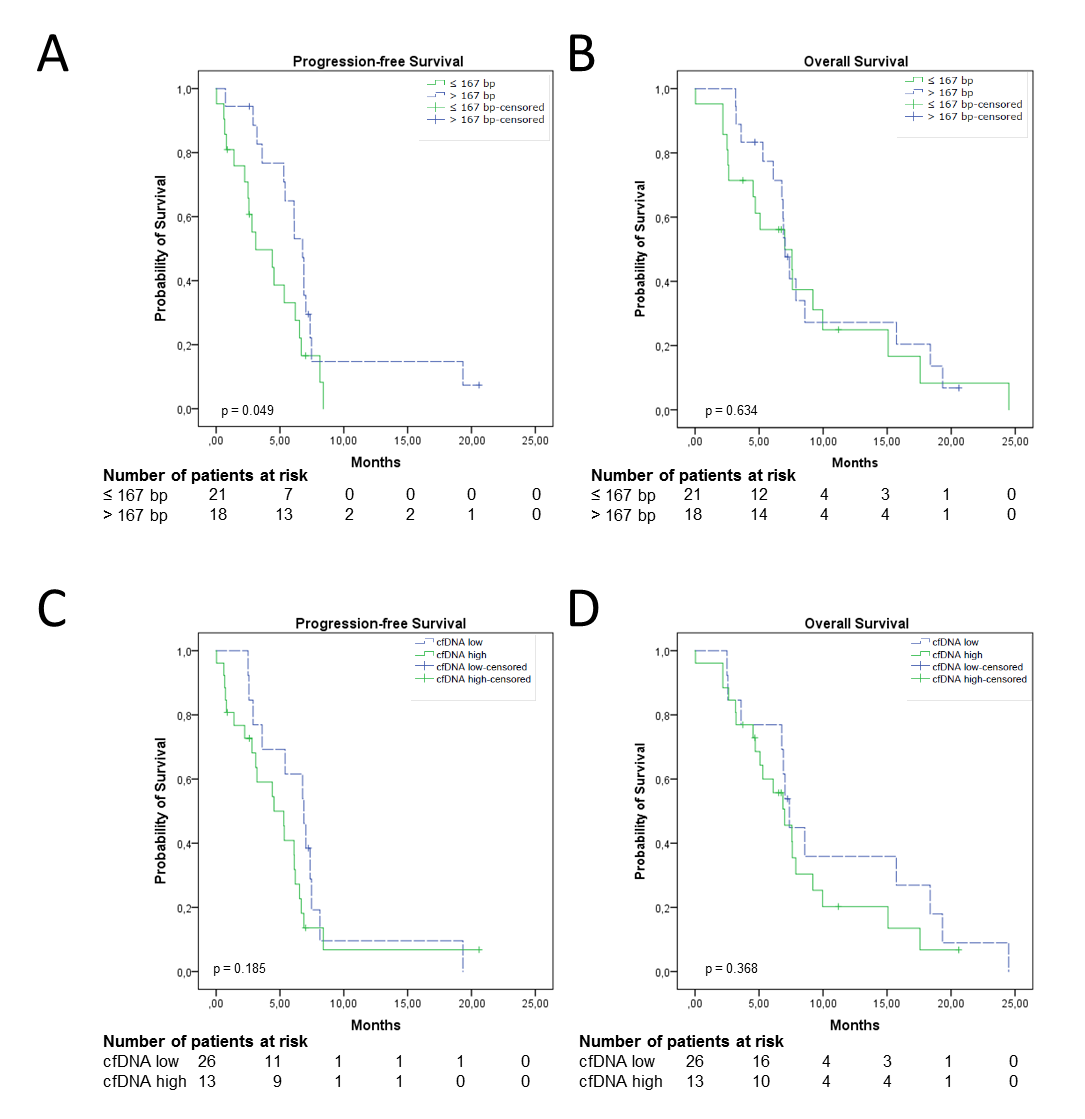
**

**Figure S4: Kaplan-Meier analyses of samples obtained after the first cycle of chemotherapy.** (**A**,**C**) Progression-free survival and (**B**,**D**) overall survival are shown for (**A**,**B**) patients with short (≤167 bp) vs. those with long (>167 bp) cfDNA fragment size, and (**C**,**D**) patients with high vs. those with low cfDNA levels.
